# Exploring the roles of intestinal flora in enhanced recovery after surgery

**DOI:** 10.1016/j.isci.2023.105959

**Published:** 2023-01-13

**Authors:** Zaoqu Liu, Na Li, Qin Dang, Long Liu, Libo Wang, Huanyun Li, Xinwei Han

**Affiliations:** 1 Department of Interventional Radiology, The First Affiliated Hospital of Zhengzhou University, Zhengzhou, Henan 450052, China; 2 Interventional Institute of Zhengzhou University, Zhengzhou, Henan 450052, China; 3 Interventional Treatment and Clinical Research Center of Henan Province, Zhengzhou, Henan 450052, China; 4 Department of Infectious Diseases, The First Affiliated Hospital of Zhengzhou University, Zhengzhou, Henan 450052, China; 5 Department of Colorectal Surgery, The First Affiliated Hospital of Zhengzhou University, Zhengzhou, Henan 450052, China; 6 Department of Hepatobiliary and Pancreatic Surgery, The First Affiliated Hospital of Zhengzhou University, Zhengzhou, Henan 450052, China; 7 Genetic and Prenatal Diagnosis Center, Department of Obstetrics and Gynecology, The First Affiliated Hospital of Zhengzhou University, Zhengzhou, Henan 450052, China

**Keywords:** Medicine, Surgery, Microbiology, Microbiome

## Abstract

Striving to optimize surgical outcomes, the Enhanced Recovery After Surgery (ERAS) pathway mitigates patients’ stress through the implementation of evidence-based practices during the pre-, intra-, and postoperative periods. Intestinal flora is a sophisticated ecosystem integrating with the host and the external environment, which serves as a mediator in diverse interventions of ERAS to regulate human metabolism and inflammation. This review linked gut microbes and their metabolites with ERAS interventions, offering novel high-quality investigative proponents for ERAS. ERAS could alter the composition and function of intestinal flora in patients by alleviating various perioperative stress responses. Modifying gut flora through multiple modalities, such as diet and nutrition, to accelerate recovery might be a complementary approach when exploring novel ERAS initiatives. Meanwhile, the pandemic of COVID-19 and the availability of promising qualitative evidence created both challenges and opportunities for the establishment of ERAS mode.

## Graphical abstract

**Figure:**
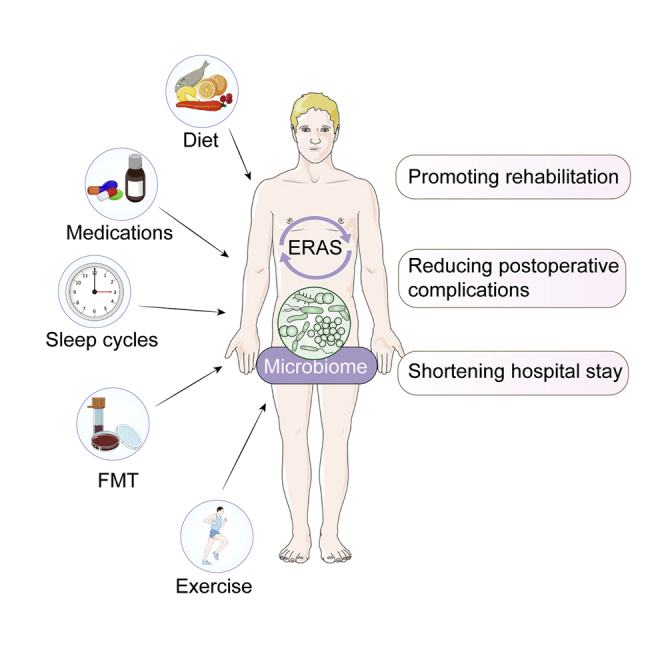

## Introduction

The past decade has marked an explosion of exciting discoveries linking the composition and function of indigenous microorganisms to human health and disease. Especially, culture-independent genomic techniques allow a comprehensive classification and evaluation of the gut microbiome, which has dramatically increased our understanding of the extensive changes in microbial composition.^1^ Association studies have documented variations in the abundance of intestinal bacteria in individuals with gastrointestinal phenotypes, including irritable bowel syndrome,^2^ inflammatory bowel disease,^3^ and colorectal cancer,^4^ as well as other system and organ disorders.^5^ Moreover, the interplay between intestinal flora and various regions in human beings is highly investigated.

Because Danish professor Kehlet first proposed the concept of fast-track surgery in the 1990s,^6^ and further developed as Enhanced Recovery After Surgery (ERAS) by the ERAS Society,^7^ the concept and approach of ERAS have been rapidly popularized and widely applied around the world. ERAS is a paradigm shift in perioperative nursing with a multi-mode/disciplinary method to care for surgical patients, which significantly improves clinical results (length of hospital stay, complications, readmissions) and saves cost on medical expenses.^8^ Nowadays, the ERAS protocol has proven to improve outcomes in almost all primary surgical specialties, including pulmonary surgery,^9^ liver surgery,^10^ gynecologic oncology,^11^ as well as head and neck cancer.^12^

ERAS could alter the composition and function of intestinal flora in patients by relieving various stress responses during the perioperative period. This affects the metabolism of the host, thus achieving the purposes of reducing postoperative complications, shortening hospital stay, and promoting rehabilitation. This review summarized the role of gut microbiota in ERAS, and how intestinal flora might regulate the metabolic processes to accelerate rehabilitation. Lastly, we explored future directions that merit consideration in basic and clinical translational studies.

## Overview of the intestinal flora

In the human gut, trillions of microorganisms (bacteria, archaea, bacteriophages, eukaryotic viruses, and fungi) coexist with human surfaces and inside every cavity of the body. The gut microbiome are nearly 1,000 more genes than in the human genome (>22 million genes in the gut microbiome as opposed to 23,000 in the human genome).^13^ Intestinal microbiota plays a paramount role in training host immunity, modifying intestinal endocrine function, regulating drug metabolism, eliminating toxins as well as generating a variety of metabolites.^14^

### Healthy gut microbiota

Generally, a healthy gut ecosystem is characterized by a diverse array of taxa, high gene richness, and stable functional cores in microbes. Despite a distinguished interindividual variation in gut microbiota composition and dynamics, it is redundant for clear persistence of conserved metabolic functions across multiple phyla. The most abundant among these core pathways include genes encoding glycosaminoglycan degradation, short-chain fatty acids (SCFAs) synthesized from complex polysaccharides, and specific lipopolysaccharides (LPS), as well as the biosynthesis of vitamins and serval essential amino acids.^15^ Table 1 showed a list of microbial fermentation products, related routes, and microbial manufacturers. It is noteworthy that the metabolism is intricately interconnected and should be viewed more like a metabolic web than as a discrete set of linear pathways.

**Table 1:** Gut bacteria and the metabolites they contribute

Metabolite	Pathway	Genera or species	Reference
Butyrate	Classical pathway via butyrate kinase	*Coprococcus comes, Coprococcus eutactus*	Macfarlane and Macfarlane, 2003, Cummings et al., 1987, den Besten et al., 2013, Smith and Macfarlane, 1997^16^^,^^17^^,^^18^^,^^19^
	Alternate pathway using exogenous acetate	*Anaerostipes spp., C. catus, E. hallii, Eubacterium rectale, Faecalibacterium prausnitzii, Roseburia spp.*	Macfarlane and Macfarlane, 2003, den Besten et al., 2013, Smith and Macfarlane, 1997, Cummings and Macfarlane, 1991^16^^,^^18^^,^^19^^,^^20^
Propionate	Acrylate pathway	*Coprococcus catus, Eubacterium hallii, Megasphaera elsdenii, Veillonella spp.*	Macfarlane and Macfarlane, 2003, Cummings et al., 1987, den Besten et al., 2013, Smith and Macfarlane, 1997^16^^,^^17^^,^^18^^,^^19^
	Succinate pathway	*Bacteroides spp., Dialister spp., Phascolarctobacterium succinatutens, Veillonella spp.*	Macfarlane and Macfarlane, 2003, Cummings et al., 1987, den Besten et al., 2013, Smith and Macfarlane, 1997^16^^,^^17^^,^^18^^,^^19^
	Propanediol pathway	*Roseburia inulinivorans, Ruminococcus obeum, Salmonella enterica*	Macfarlane and Macfarlane, 2003, Cummings et al., 1987, den Besten et al., 2013, Smith and Macfarlane, 1997^16^^,^^17^^,^^18^^,^^19^
Acetate	Pyruvate decarboxylation to acetyl-CoA	*Akkermansia muciniphila, Bacteroides spp., Bifidobacterium spp., Prevotella spp., Ruminococcus spp*	Macfarlane and Macfarlane, 2003, Cummings et al., 1987, den Besten et al., 2013, Smith and Macfarlane, 1997^16^^,^^17^^,^^18^^,^^19^
	Wood–Ljungdahl pathway	*Blautia hydrogenotrophica, Clostridium* spp.*, Streptococcus* spp.	Macfarlane and Macfarlane, 2003, Cummings et al., 1987, den Besten et al., 2013, Smith and Macfarlane, 1997^16^^,^^17^^,^^18^^,^^19^
Branched-chain fatty acids	Amino acid fermentation through various dissimilatory proteolytic reactions	*Acidaminococcus spp., Acidaminobacter spp., Campylobacter* spp.*, Clostridia spp., Eubacterium spp., Fusobacterium spp., Peptostreptococcus spp.*	Macfarlane and Macfarlane, 2003, Cummings et al., 1987, den Besten et al., 2013, Smith and Macfarlane, 1997, Deehan et al., 2020^16^^,^^17^^,^^18^^,^^19^^,^^21^
Imidazole propionate	Non-oxidative deamination of histidine to urocanate followed by reduction of urocanate to ImP by urocanate reductase	*Aerococcus urinae, Adlercreutziae equolifaciens, Anaerococcus prevotii, Brevibacillus laterosporus, Eggerthella lenta, Lactobacillus paraplantarum, Shewanella oneidensis, Streptococcus mutans*	Koh et al., 2018^22^
Indole	Hydrolytic β-elimination of tryptophan to indole (tryptophanase)	*Achromobacter liquefaciens, Bacteroides ovatus, Bacteroides thetaiotamicron, Escherichia coli, Paracolobactrum coliforme, Proteus vulgaris*	Devlin et al., 2016, Agus et al., 2018^23^^,^^24^
Indole derivatives	Multiple	*Bacteroides spp., Clostridium* spp. *(Clostridium sporogenes, Clostridium cadaveris, Clostridium bartlettii), E. coli, Lactobacillus spp., E. halli, Parabacteroides distasonis, Peptostreptococcus spp. (Peptostreptococcus anaerobius)*	Devlin et al., 2016, Agus et al., 2018, Dodd et al., 2017^23^^,^^24^^,^^25^
Trimethylamine N-oxide	Intestinal bacteria metabolize choline, choline-containing compounds, betaine, and L-carnitine ingested in the diet or recycled in the gut	*Desulfovibrio desulfuricans, Acinetobacter, Serratia, Klebsiella pneumoniae, E. coli, Citrobacter, Providencia, Shigella, Achromobacter, Sporosarcina, Actinobacteria, Edwardsiella tarda*	Craciun and Balskus, 2012, Thibodeaux and van der Donk, 2012, Falony et al., 2012, Romano et al., 2012^26^^,^^27^^,^^28^^,^^29^

**Bile acids**			

Primary bile acids	Synthesized in the liver from cholesterol via classical or alternative pathways and bound to taurine or glycine	Host (mouse and human)	Wahlström et al., 2016, Jia et al., 2016^30^^,^^31^
Secondary bile acids	Deconjugation of primary and secondary bile acids through bile salt hydrolases	*Bacteroides, Bifidobacterium, Clostridium, Eubacterium, Lactobacillus*	Wahlström et al., 2016, Jia et al., 2016^30^^,^^31^
	Conjugation to phenylalanine, tyrosine or leucine	*Clostridium bolteae*	Quinn et al., 2017^32^
	7α/β-dehydroxylation; 3α/β-epimerization; 5β/α-epimerization; 6β-epimerization of β-MCA; 7α/β-epimerization of CDCA and β-MCA; oxidation of primary or secondary bile acids at C3, C7, and C12	*Bacteroides, Clostridium, Escherichia, Eubacterium, Lactobacillus; Eubacterium lentum, Clostridium perfringens, Ruminococcus gnavus; Eubacterium; Eubacterium, Fusobacterium,* Unidentified Gram-positive rod; *Clostridium,* Unidentified Gram-positive rod; *Bacteroides, Clostridium, Eggerthella, Escherichia, Eubacterium, Peptostreptococcus, Ruminococcus*	Wahlström et al., 2016, Jia et al., 2016, Funabashi et al., 2020, Eyssen et al., 1983, Demarne et al., 1982^30^^,^^31^^,^^33^^,^^34^^,^^35^

### Structural constituents and metabolites of bacteria regulate host metabolism

**Figure 1:**
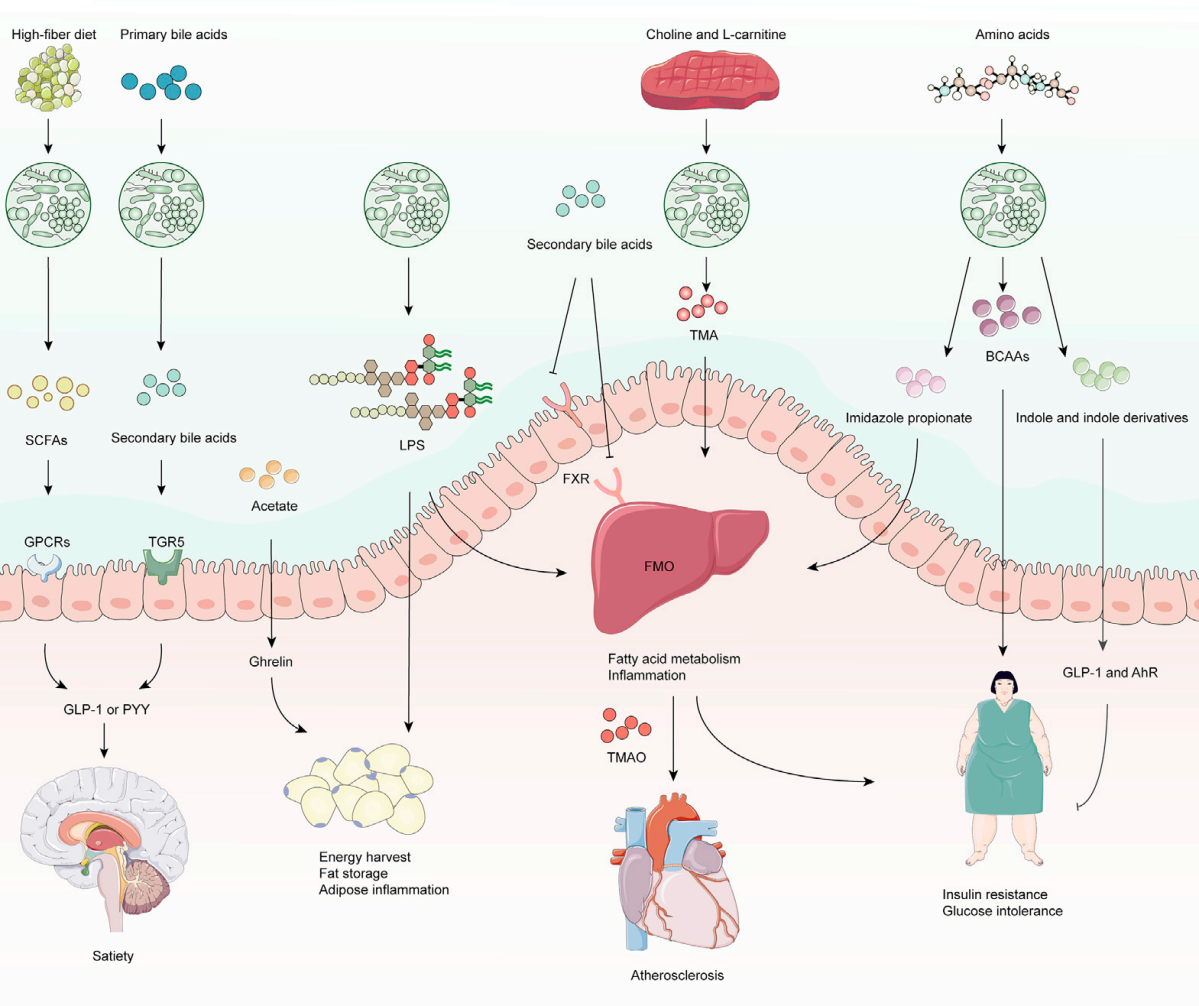
Gut microbiota and metabolites regulate host metabolism. Several gut microbial compounds influence glucose tolerance, homeostasis, insulin sensitivity, lipid metabolism, triglyceride and cholesterol levels, as well as energy expenditure Dietary fiber-derived SCFAs directly activate GPCRs and stimulate the release of GLP-1 (insulin biosynthesis) and PYY (satiety). In addition, acetate enhances fat storage by inducing the secretion of ghrelin. Bacterial LPS are liberated upon bacterial cell death and serves as virulent stimulators of host immunity. Bile acids regulate glucose and lipid metabolism via FXR (impairment) and TGR5 (facilitation). TMA from dietary choline and L-carnitine sources is oxidized in the liver by FMO to TMAO, which is responsible for atherosclerosis. Imidazole propionate, a bacterial metabolite derived from histidine, contributes to insulin resistance. Elevated circulating concentrations of BCAAs are powerful biomarkers of insulin resistance and increased risk of T2D. Indole and its derivatives prevent chronic inflammation, hepatic steatosis and insulin resistance by targeting AhR ligands or GLP-1.

The physiological and metabolic properties in both healthy and diseased states are inevitably affected by the intestinal flora. The first evidence for the mechanistic involvement of intestinal flora in metabolic regulation was shown in 2004, where it was found that intestinal flora regulated the host’s acquisition and storage of energy from the diet in rats.^36^ Given the essential importance of metabolite measurements for understanding the host-microbe relationship and the emerging value of the epigenome as a sophisticated signaling integrator, extensive new knowledge on the prospective role of gut microbiota in metabolism has been furnished.^37^ In the following sections, several gut microbial components impacting glucose homeostasis, insulin sensitivity, lipid metabolism, and host energetic expenditure were discussed (Figure 1).

SCFAs butyrate, propionate, and acetate, primarily fermented from complex carbohydrates (for instance, fiber), might be metabolically protective. Specifically, SCFAs activate G protein-coupled receptors (GPCRs) and inhibit histone deacetylases, which are the principal energy sources for intestinal epithelial cells.^38^ Propionate and butyrate have anti-obesity effects predominantly through stimulating the synthesis of anorexigenic hormones and leptin. Butyrate also facilitates the polarization of colonic anti-inflammatory regulatory T cells to suppress inflammatory responses, and induces NOD-, LRR- and pyrin domain-containing protein 3 (NLRP3) inflammasomes via the GPCRs approach.^39^ In addition, direct activation of olfactory receptor 78, which regulates blood pressure, is also possible through acetate and propionate.^40^ As opposed to butyrate and propionate, acetate might be predominantly obesogenic. Fat storage could be facilitated by acetate because it stimulates ghrelin secretion.^41^ It is reported that the intestinal hormones, peptide YY (PYY) and glucagon-like peptide 1 (GLP-1) were induced by rectal or intravenous administration of acetate.^42^ Sanna et al. and Tirosh et al., nonetheless, have lately revealed conflicting results regarding the effects of propionate and butyrate on host metabolism.^43^^,^^44^ These contradictory experimental outcomes require additional human investigations of each SCFA individually or in combination depending on its proportional concentration in circulation.

Various tissues respond to bile acids that regulate lipid and glucose metabolism as well as inflammation. Primary bile acids are synthesized in hepatocytes from cholesterol and transformed into secondary bile acids by intestinal flora.^45^ Both categories of bile acids modulate the integration via FGF19/FGF15,^46^ whereas they do take on metabolic effects through the nuclear receptor, farnesoid X receptor (FXR), and the membrane G protein-coupled receptor, TGR5. The opposite directions of the signaling system are involved with glucose and lipid metabolism, which FXR impairs^47^ and TGR5 promotes.^48^ Nevertheless, the FXR-dependent role of secondary bile acids in the glycolipid metabolism is contextually relevant. In regular diet FXR-deficient mice, accumulation of hepatic lipids, triglycerides, and cholesterol was noticed,^49^ whereas, in mice on the high-fat diet (HFD), deficiency of FXR enhanced glucose equilibrium and decreased body mass,^50^ presumably as a result of different basal gut flora.

The branched-chain amino acids (BCAAs), leucine, isoleucine, and valine represent among the nine essential amino acids which are manufactured by intestinal bacteria. Elevated circulating concentrations of BCAAs are powerful biomarkers of insulin resistance and type 2 diabetes (T2D).^51^ BCAAs catabolism is essential for the control of thermogenesis in brown adipose tissue (BAT). It contributes to the amelioration of metabolic status through the SLC25A44 transporter protein that occurs in mitochondria.^52^ Of interest, Zhou et al. revealed that the accumulation of BCAAs induced insulin resistance in genetically mice with obesity.^53^ Correspondingly, in insulin-resistant patients, there was a decrease in the abundance of bacteria competent to absorb BCAAs, such as *Butyrivibrio crossovers* and *Eubacterium siraeum*.^54^

Intestinal microbiota metabolizes dietary nutrients such as L-carnitine, phosphatidylcholine, and choline, to generate trimethylamine (TMA), which is assimilated in the gut and turned into trimethylamine-N-Oxide (TMAO) by flavin-containing monooxygenase 3 in the liver.^55^ Supplementation of the mouse diet with TMAO, carnitine, or choline could modify the microbial composition in the cecum, contributing to the production of TMA/TMAO, which enhances the risk of atherosclerosis.^56^ Overall, TMAO drives inflammation and platelet activation, thereby correlating with atherosclerosis, cardiovascular complications, and clinical outcomes.

Indole and its derivatives are synthesized directly from dietary tryptophan by the metabolism of intestinal microorganisms. Several of them serve as aryl hydrocarbon receptor (AhR) ligands. Emerging evidence indicates that reduced microbial capacity to produce AhR activating metabolites might be a crucial element of the metabolic syndrome. The mechanism could be related to gut permeability and LPS transposition caused by diminished secretion of GLP-1 and IL-22, which is responsible for chronic inflammation, insulin resistance, and hepatic steatosis.^57^ Analogously, epidemiological investigations have reported that circulating concentrations of 3-indolepropionic acid (IPA) were correlated with enhanced insulin sensitivity and decreased risk of T2D.^58^

In conclusion, structural constituents and metabolites released by bacteria that deal with both dietary and host-derived molecules regulate metabolic inflammation via myriad regimes. This raises prospective targets for exploring innovative therapeutic vehicles for metabolic diseases. It is noteworthy that metabolic inflammation and gut microbiota assembly are dominated by other parameters. Modifications in the intestinal flora could moderate and perpetuate the constitutive health of the organism, which establishes the foundation for a range of interventions in ERAS.

## Linkages between ERAS and intestinal microecology

**Figure 2:**
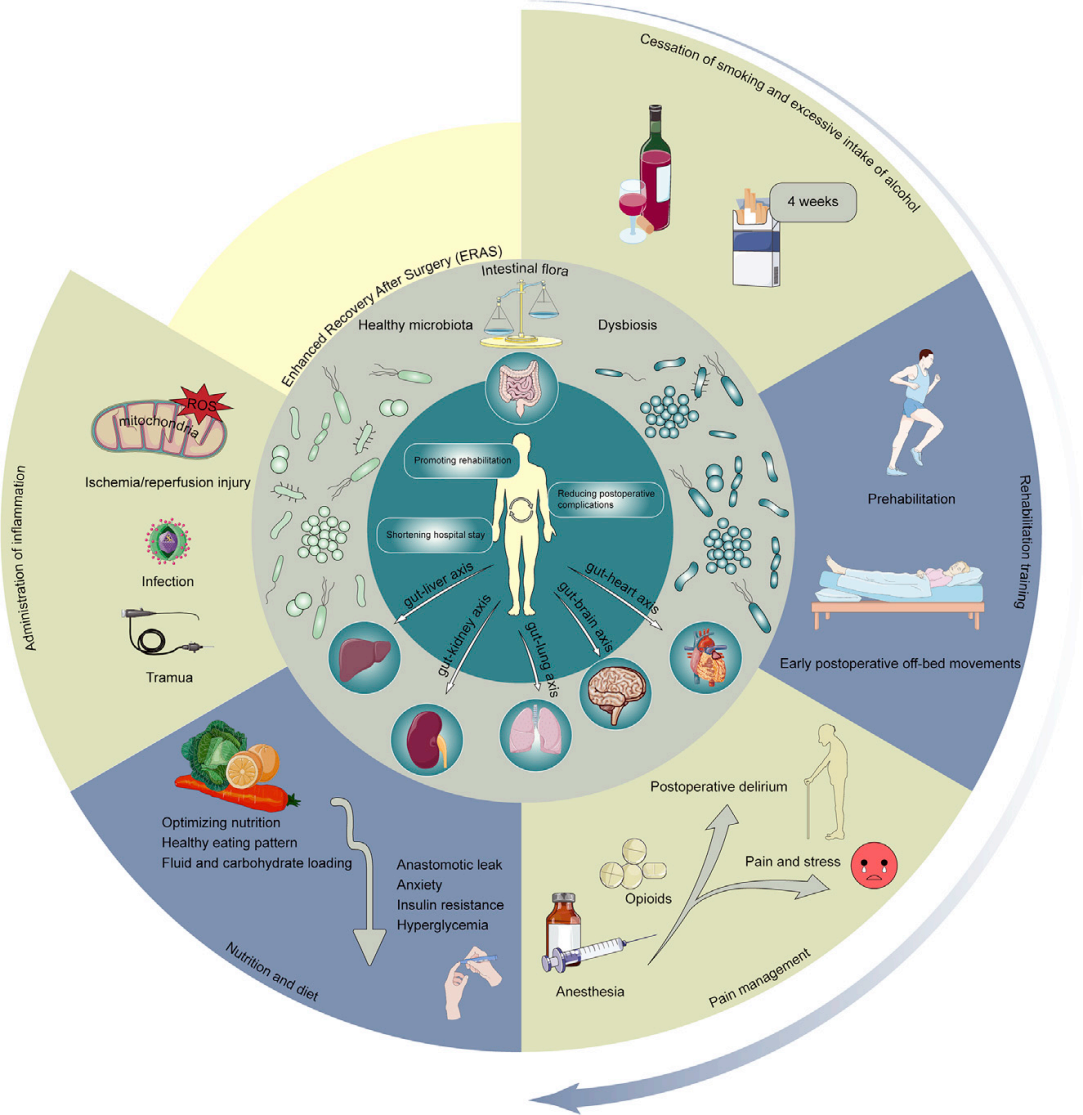
Linkages between ERAS and intestinal microecology. The microbiota has been categorized as the largest “endocrine organ” in the human body It can regulate all tissues and organs of the body through gut-liver axis, gut-kidney axis, gut-lung axis, gut-brain axis and gut-heart axis. The multiple pathways of ERAS can coordinate the shift toward healthy intestinal flora, thereby reducing postoperative complications, shortening hospital stay and promoting rehabilitation. It was demonstrated that preoperative smoking and alcohol cessation for more than 4 weeks significantly diminished the overall complication rate, which might be correlated with the contribution of intestinal flora. A preoperative campaign regimen and early postoperative off-bed activities can facilitate restoration of multisystem function. Appropriate pain management in the perioperative period effectively reduces the stress response and promotes early recovery of bowel function in patients. Dietary interventions can decrease the incidence of postoperative anastomotic fistula. On one hand, it may alleviate patient anxiety, whereas on the other hand, it may directly act on intestinal flora to reduce postoperative insulin resistance and hyperglycemia. Furthermore, intestinal flora plays an instrumental role in the regulation of excessive inflammatory responses because of multiple factors in the perioperative period, including trauma, infection, and intraoperative I/R injury. In conclusion, various interventions in ERAS are available to attenuate perioperative patient irritation by modulating the intestinal flora and its metabolites, thus enhancing recovery.

Currently, the microbiota has been categorized as the largest “endocrine organ” in the human body.^59^ This “organ” is modifiable, and the configuration and dosage of the microbiota could be manipulated through nutrition and pharmaceuticals to influence health. The individual diversification of intestinal flora coincides with the patient-oriented “personalized” treatment concept advocated by the principle of ERAS. Emerging evidence highlighted vital synergies between the gut microbiota and extracutaneous organs, including the lung (gut-lung axis),^60^ liver (gut-liver axis),^61^ and CNS (gut-brain axis),^62^ etc. Correspondingly, ERAS could be extended to surgeries on systemic organs. The multiple pathways of ERAS are committed to alleviating the stimulation of perioperative patients, and harmonize the intestinal flora and its metabolites, thereby facilitating recovery (Figure 2).

### The concept of ERAS: Preoperative, intraoperative, postoperative

ERAS is implemented on the foundation of evidence-based medicine and optimizes clinical trajectories involving perioperative interventions through multidisciplinary collaboration among surgery, anesthesia, nursing, nutrition, etc. Kehlet et al. first discovered that in elderly high-risk patients receiving colonic surgery, substantial progress in postoperative recovery could be accomplished through early and aggressive perioperative care.^6^ Subsequently, ERAS Society proposed the ERAS protocol: a multidisciplinary team approach focused on reducing stress and facilitating functional recovery, aiming at a faster recovery from major surgery, avoiding the medium-term sequelae of conventional postoperative care, and minimizing health care costs by shortening hospital stays.^7^ Surveys have revealed that the implementation of ERAS could cut postoperative complications by 50%, shorten hospital admissions by 30%, and slash readmission rates, thereby trimming healthcare costs.^63^ Table 2 outlined the core interventions and elements of ERAS typically utilized in this process. Generally, ERAS comprise several elements that have a collective focus: they miniaturize stress and refine the response to stress.^8^ By strengthening physiological reserves and enhancing functional capacity, patients eschew catabolism. Insulin resistance is diminished during tissue damage, thus obviating the loss of protein, muscle strength, and cellular fitness.

**Table 2:** Core elements and interventions of ERAS

Preoperative	Intraoperative	Postoperative
Smoke cessation; Alcohol abstinence	Standardized anesthesia, avoiding long-acting opioids; Epidural anesthesia for open surgery	Early intake of oral fluids and solids
Rehabilitation training	Minimal invasive surgical techniques	Intake of protein and energy-rich nutritional supplements
Psychological intervention	Effective management of inflammation	Multimodal approach to control of nausea and vomiting
Optimizing nutrition	Maintaining fluid balance; Administer vasopressors to support blood pressure control	Support ambulation and mobilization
Fluid and carbohydrate loading; No prolonged fasting	Lung protection	Non-opioid oral analgesia/NSAIDs
No/selective bowel preparation	Brain protection	Patient blood management (PBM)
Antibiotic prophylaxis	Maintenance of normothermia (body warmer/warm intravenous fluids)	No nasogastric tubes
Pain management	Prevention of stress hyperglycemia	Early removal of the catheter
Thromboprophylaxis	No drains	Audit of compliance and outcomes
Correction of preoperative anemia	–	–

### Cessation of smoking and excessive intake of alcohol

Smoking compromises microbial community function, especially the metabolism of amino acids, carbohydrates, propanoate, and indoles. It was demonstrated that smoking cessation for more than 4 weeks before surgery significantly shortened the postoperative hospital stay and reduced the rate of overall complications.^64^ This might be relevant to the alteration of intestinal flora diversity by smoking and the increase in the abundance of pro-inflammatory bacteria such as *Ruminococcus gnavus*, *Bacteroides vulgatus*, etc.^65^ Dysbiosis of the intestinal flora destabilizes metabolic homeostasis and leads to insulin resistance. A recent study by Lai et al. isolated a symbiotic bacterium, *Parabacteroides goldsteinii*, that ameliorated smoking-induced lung disease via the gut-lung axis.^66^ Nevertheless, only a few investigations have explored the effects of smoking cessation on intestinal flora in smokers, and they have presented ambivalent outcomes.^67^^,^^68^^,^^69^ Whether the health benefits of quitting smoking in the short term would encompass salutogenic alterations in the gut flora is unclear.

Likewise, 4 weeks of preoperative abstinence from alcohol significantly decreases the incidence of postoperative complications whereas 2-week withdrawal from alcohol markedly enhanced platelet function and shortened bleeding time.^70^ The pathological influences of alcohol on the intestinal flora are partially contingent on the gut-liver axis.^71^ For most patients with moderate to severe alcohol use disorder (AUD), alcohol withdrawal is an aggressive gut microbial intervention and a key to precluding further organ damage.^72^ Markers of intestinal barrier function, such as overall TLR4/TLR2 ligands in serum, also dramatically ameliorated after merely one week of abstinence.^73^ Nevertheless, whether the multiple adverse behavioral responses such as negative emotions arising from abrupt withdrawal in AUD patients would impinge on clinical outcomes demands further investigation.

### Pain management

The intestinal microbiota exerts a decisive role in diverse categories of chronic pain, especially visceral pain. Appropriate pain manipulation in the perioperative period could efficiently diminish stress and insulin resistance, as well as facilitate the early restoration of intestinal function in patients.^8^ Inequivalent postoperative pain control is correlated with perioperative morbidity, including myocardial infarction and pulmonary complications. Microorganisms in the gut manufacture metabolites, neurotransmitters, and neuromodulators that interoperate with receptors to regulate peripheral and central sensitization implicated in chronic pain.^74^^,^^75^

Anesthesia and surgery could provoke perioperative neurocognitive dysfunction (PND), which is probably attributable to modifications in intestinal microecology. The administration of certain general anesthetics could result in a depletion of microbiome diversity as well as neurologically relevant metabolite variations.^76^ In contrast, continuous intravenous infusion of propofol, a short-acting anesthetic recommended by ERAS, had no measurable influence on the intestinal flora of rats.^77^

Prolonged opioid treatment has been linked to microbial imbalances among humans and mice. Low-opioid multimodal analgesic strategy in ERAS could mitigate the detrimental effects of opioids on anesthetic awakening and postoperative bowel functions. Opioids administered for pain management have been demonstrated to induce expansion of pathogenic bacteria, bacterial translocation, immune dysregulation, and sustained inflammatory responses.^78^ Similarly, methadone maintenance therapy (MMT) could trigger an imbalance in the generation of SCFAs, mucus degradation, and critical bacterial communities required for the sustenance of barrier integrity.^79^ Shakhsheer et al. demonstrated that morphine facilitated the tissue colonization of collagenolytic *Enterococcus faecalis* which contributed to the anastomotic fistula.^80^ In addition, morphine suppressed the outgrowth of intestinal organoids from HIV-transgenic mice by dampening Notch signaling in a histone deacetylases (HDAC)-dependent manner.^81^ Notably, opioids that agonize predominantly κ receptors have milder adverse effects in terms of intestinal paralysis and postoperative nausea and vomiting while being effective in attenuating surgically provoked visceral pain. Currently, there is a lack of investigations to delineate whether the discrepancies in the adverse effects of opioid drugs agonizing different receptors are affiliated with intestinal flora.

### Nutrition and diet

Dietary changes could optimize surgical outcomes through effects on the gut microbiota. ERAS recommends preoperative oral carbohydrate beverages, nutritional condition improvement, and early postoperative reinstatement of transoral diet. Hyoju et al. have revealed that a short-term low fat/high fiber diet could prevent the detrimental impact of long-term HFD feeding on intestinal flora and anastomotic healing in mice.^82^ Preoperative carbohydrate drinks could alleviate preoperative anxiety and minimize the incidence of postoperative insulin resistance and hyperglycemia, thereby significantly curtailing the length of stay (LOS).^83^ Early postoperative resumption with oral feeding and drinking could facilitate recovery of intestinal function, support preservation of the intestinal mucosal barrier, and preclude migration of the flora.

Intestinal flora serves an imperative role in dietary interventions to mitigate postoperative insulin resistance and catabolism. A dietary intervention study revealed a considerable increment of microbial pathways producing acetate and butyrate in patients with T2D after 12 weeks of consumption on a diet rich in combinatorial fiber mixture.^84^ This coincided with an improvement in circulating PYY levels under fasting conditions and GLP-1 levels in postprandial conditions. Furthermore, certain proteolytic fermentation products have also appeared to be engaged in the pathogenesis of insulin resistance.^54^ It is worth noting that diabetic patients are at risk for poor perioperative glycemic control or pulmonary aspiration and that patients with type 1 diabetes are insulin deficient, not insulin resistant. These effects warrant further confirmation in a larger cohort of human intervention studies, and the underlying mechanisms demand an investigation.

### Administration of inflammation

Abiogenesis intrinsic taxa are engaged in ERAS to mediate the host response to inflammatory triggers, whereas the inflammatory reaction conversely exacerbates intestinal flora dysbiosis. Excessive inflammatory responses could originate during the perioperative period for manifold factors, including trauma, infection, intraoperative ischemia/reperfusion (I/R) injury, as well as imbalance in systemic oxygen supply and demand because of improper anesthetic management or circulatory instability. Perioperative inflammation management interventions in ERAS could optimize postoperative regression and long-term survival of patients.^85^ We elaborated on the potential regulatory mechanisms and interventions concerning intestinal flora based on the example of I/R injury in multiple organs.

A distinctive bifurcated correlation exists between intestinal flora and I/R injury.^86^ The increase in Enterobacteriaceae and the decrease in *Lactobacillus* and *Ruminococcaceae* were signatures of ecological misalignment because of I/R injury and were accompanied by a decrement in SCFAs levels, intestinal inflammation, and enteric leakage.^87^ Nonetheless, the contribution of intestinal flora and its metabolites to the I/R injury remained polemical. The gut microbial metabolite capsiate has been investigated to mitigate intestinal I/R damage by activating TRPV1, enhancing Gpx4 expression, thereby inhibiting ferroptosis.^86^ The intestinal microbial metabolite pravastatin facilitated IL-13 release from type II innate lymphoid cells via the IL-33/ST2 signaling pathway, which attenuated intestinal I/R injury.^88^ Butyrate treatment could attenuate I/R-induced renal damage by upregulating intracellular oxidative stress and inflammation.^89^ Similarly, in the brain and myocardial I/R injury, several intestinal flora metabolites have been reported to exert the inimitable protective properties through specific signaling pathways.^90^ In addition, probiotics might alleviate I/R and the distal organ lesion through anti-oxidative stress as well as an anti-inflammatory response.^91^ Nevertheless, in contrast to preliminary findings, an oral antibiotic to mitigate microbiota had a beneficial impact on I/R injury. This nephroprotective effect correlated with the decrease in Th17 and Th1 responses with the dilatation of regulatory T cells and M2 macrophages.^87^ Corroborating with this result, mice with declined intestinal flora expressed lower levels of F4/80 and chemokine receptors CX3CR1 and CCR2 than in control mice, with a promising profile against renal I/R injury.^92^ This presumably accounts for the dramatic changes in the organic environment under ischemic and hypoxic conditions, upregulating pathogenic factors, and inflammatory responses. To summarize, intestinal flora intermediates the anti-stress and inflammatory control measures in ERAS and constitutes promising intervention targets.

Moreover, ERAS recommended that all patients undergoing elective colorectal surgery should be considered for oral antibiotics (OAB). A multicenter, pragmatic, randomized controlled trial demonstrated that OAB prophylaxis the day before colon surgery could remarkably decrease the incidence of surgical site infections.^93^ Nevertheless, the problem of OAB effect on intestinal flora is complicated by variations in the antibiotic treatment administered, the subjects treated, and the specific methods employed. For instance, experimental and clinical evidence indicated that rifaximin might exert beneficial effects on the cirrhotic process by modulating the intestinal microbiome and shaping the gut-liver axis.^94^ Rifaximin also attenuated Pregnane X Receptor-dependent toxicity induced by *Clostridium difficile toxin A* in Caco-2 cells via TLR-4 pathway.^95^ However, Kang et al. identified that rifaximin modified intestinal ammonia production by regulating the expression of intestinal glutaminase independent of gut microbiota.^96^ The evidence to date is grounded in cross-sectional studies or smaller longitudinal investigations with varying results not only for host effects but also for microbiome effects. It is apparent that examining the detailed mechanisms of how OAB influences the human gut microbiota is a worthwhile endeavor for ERAS strategies.

## Intestinal flora-mediated potential interventions for ERAS

**Figure 3:**
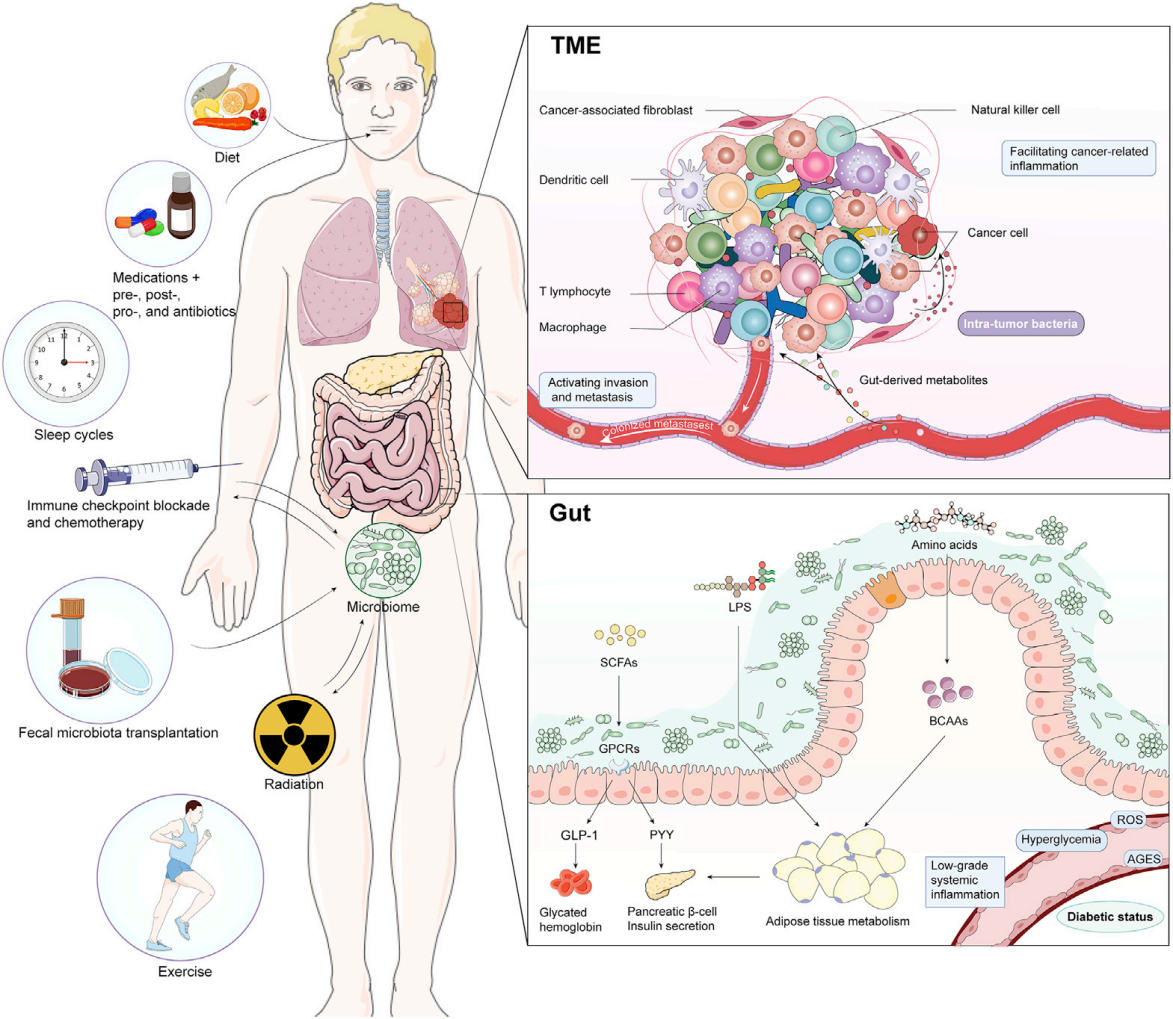
Targeting microbes to formulate individualized ERAS measures for T2D and oncology patients ERAS initiatives that can be considered, such as diet, medications, probiotics, fecal microbiota transplantation, exercise and sleep cycles all have the ability to alter the microbiomes and may affect patient prognosis. Gut microbial-derived mechanisms are able to regulate the chronic inflammatory state in T2D. Dietary fiber enhances the production of SCFAs and influences glycated hemoglobin levels and pancreatic insulin secretion via GLP-1 or PYY. LPS and protein-derived BCAAs can modify adipose tissue metabolism and gut microbiome composition. Gut and tumor microenvironment (TME) microbiomes regulate host metabolism and immunity, which ultimately influence anti-tumor immunity. Metabolites, toxins and antigens generated by microbiota impact tumor proliferation, inflammation, cell signaling and metastasis. Moreover, there may be a bidirectional interaction between these microbiota and cancer treatments (radiotherapy, chemotherapy and immunotherapy).

Microbiota, comprising hundreds of bacterial strains, constitute an ecological network with more or less favorable status, which could be a formidable way to fulfill the commitment of precision medicine. Therefore, we envisioned targeting the unique microorganisms in each individual to avail personalized interventions for ERAS, especially in those patients suffering from metabolic and tumor diseases, which are intimately intertwined with intestinal flora (Figure 3).

### Type 2 diabetes

Permissible techniques to modulate the microbiome comprise dietary interventions, probiotics or prebiotics, bacterial consortia, and fecal microbiota transplantation (FMT). Among them, diet, a key determinant of microbial community structure, is the most modifiable and relatively innocuous factor for perioperative patients. It has been established that personalized diets could successfully modify postprandial blood glucose elevation and consistent alterations to gut microbiota configuration.^97^ A randomized prospective study has identified diet-specific influences of high-fermented or high-fiber foods on microbiome and immunity, rendering diet an intriguing candidate for curative interferences.^98^ In patients with T2D, who are at risk for inadequate perioperative glycemic management and insulin resistance, dietary interventions are extremely recommended. Dietary fiber facilitated a selective group of SCFAs-producing strains, contributing to the amelioration of hemoglobin A1c levels in participants, partly because of incremental generation in GLP-1.^84^ Clinical data have illustrated that incrementing the availability of non-digestible but fermentable carbohydrates were sufficient to elicit clinically relevant metabolic improvements.^84^ Mediterranean diet (MD) enabled enhanced bacterial abundance, which could better control glycemia in subjects with T2D.^99^ However, in a 21-day randomized controlled hospitalized crossover feeding trial of 20 insulin-resistant women with obesity, those on a high protein diet had superior modifications of insulin resistance and glycemic variability compared with MD.^100^ In addition, a randomized, placebo-controlled, double-blind, crossover trial revealed that the reduction of BCAAs in short-term diets diminished postprandial insulin production and modified the metabolism of white adipose tissue as well as intestinal microbiome composition.^101^ Notably, the persistence or extinction of microbiota following a specific diet ought to be considered when devising effective microbial targeting regimens. There remains a lack of research exploring whether the integration of these dietary measures targeting intestinal flora into ERAS protocols for T2D patients may improve prognosis.

### Anti-tumor therapy

The implementation of the ERAS strategy should be not only constrained to surgery but also integrated into the treatment of patients with malignancies to enhance prognosis and survival. Intestinal flora influences the mobilization of the immune system, facilitate cancer-related inflammation, and eventually affect the tumor response to therapy. Gut microbiota serves as a reservoir factor in moderating the anti-tumor immune response to chemotherapy,^102^ radiotherapy,^103^ and immune checkpoint blockade^104^ has recently been reinforced. In two recent articles, Baruch et al. and Davar et al. proposed the first demonstration on the clinical utility of FMT transfer in patients with metastatic melanoma resistant predominantly to immune checkpoint blockade.^105^^,^^106^ However, the long-term efficacy and stability of FMT remain unknown. A retrospective cohort study revealed correlations between the components of the fecal microbiome and the clinical outcomes of individuals administered CD19 CAR T-cell immunotherapy.^107^ Antibiotics appeared to abrogate the immunotherapeutic response by suppressing the gut microbiome.^108^ Exposure to antibiotics before infusion of CAR T cells was linked to lower survival and elevated toxicity in patients with B-cell malignancies.^107^ Paradoxically, however, antibiotics enhanced immunotherapeutic efficacy by upregulating PD-1 expression during the elimination of the microbiome within pancreatic tumors.^109^ Other factors that regulate the components of gut microbiota, including diet,^110^ sleep cycles, exercise, and medications, might also be incorporated into ERAS interventions to enhance anti-tumor immunity, with many epidemiological associations but few causal mechanisms. Additional clinical trials are warranted to examine how to design valid ERAS protocols for oncology patients.

Furthermore, there is accumulating evidence that microorganisms are also integral to the tumor tissue itself, with potential bacterial transfer from the intestine to the peritumoral milieu.^109^ The reorganization of microbiota in tumor-bearing mice with patients' feces revealed that immune cells were recruited or absent in the tumor environment and influenced tumor growth, marking a tremendous curative potential in manipulating microbiota to raise the life expectancy for patients.^111^ Several studies in lung, colon, and pancreatic cancer have documented that elimination of intra-tumor microbiota could restrain pro-tumor inflammatory processes, reduce cell proliferation, or convert a tolerogenic tumor microenvironment (TME) to an immunogenic one.^109^^,^^112^^,^^113^^,^^114^ In addition, the latest evidence demonstrated that tumor-resident microbiota played a significant role in accelerating tumor metastasis.^115^ Carcinoma cells could use intracellular microbiota to survive under fluid shear stress in the circulatory system during metastatic colonization.^115^ Nonetheless, whether the gut microbiome and immune system cooperate with intra-tumor bacteria to dictate cancer progression remains an unresolved matter. Modulation of the gut and/or tumor microbiota might be a promising strategy to optimize oncology therapy, which prompts new orientations for the development of ERAS.

## Conclusion and perspective

This was the original collaboration between ERAS and intestinal microecology, which indicated that there was still a substantial gap in this field. The mediating ramifications of intestinal flora in the partial ERAS measures were synthesized, gaining novel insights into ERAS. However, challenges and controversies might also emerge with the lapse of time and the availability of new high-quality evidence. For instance, the ERAS guidelines recommended against the utilization of mechanical bowel preparation (MBP) in colonic operations. Nevertheless, a thorough meta-analysis recently demonstrated that the coadministration of OAB with MBP dramatically lowered the prevalence of global complications without an increase in *C. difficile* infection compared to MBP alone.^116^ This observation necessitates high-quality randomized controlled trials to investigate the equivalence of MBP combined with OAB versus OAB alone, with consideration of microbial homeostasis. Over the forthcoming phase of ERAS, there remains an urgent aspiration for expeditious, low-cost, and qualitative research to fulfill the void in this domain.

The COVID-19 pandemic has brought unmissable opportunities and challenges for ERAS evolution. Formulating adequate perioperative nursing care for patients in need of acute and intensive caution related to COVID-19 infection is an urgent task. Gut microbiota might contribute to the magnitude of COVID-19 by registering the host immune response,^117^ which opens up the prospect of gut bacterium-mediated interventions in ERAS. It is worth noting that prophylaxis with COVID-19 has been demonstrated to transform the intestinal flora, thereby mediating pathogen susceptibility and nosocomial infections.^118^ Furthermore, COVID-19 catalyzed the advancement of Internet healthcare, especially telemedicine. Medical centers are now confronting COVID-19 through the rapid deployment of digital tools and technologies such as telemedicine and virtual care, which refers to the delivery of digital or telemedicine services to patients utilizing information and communication technologies. This is on the occasion of modernizing perioperative care interventions for ERAS, establishing contemporary monitoring and auditing by multidisciplinary teams with a view to optimizing patient prognosis.

Currently, most investigations covering the interactions between ERAS, microbiome, and human physiology remained pertinent, whereas only a few studies have delineated the underlying mechanisms of these three entities. In human experiments, a considerable group of participants is required to surmount the tremendous individualized variability among microbiomes. Moreover, functional research on bacterial metabolism are generally implemented in monocultures *in vitro*, which are unable to reproduce the actual milieu of the gut, and hence neglect the cross-feeding network constituted by the intestinal microbiota and the host response. Notwithstanding these limitations, the availability of large and comprehensive datasets as well as the employment of computational tools portend a promising scenario for microbiome exploration. Over the forthcoming decade, we anticipated that preclinical and pertinent clinical studies would offer exhilarating new insights into how ERAS moderates immunometabolic phenotypes in host and distant tissues via the gut flora, leading to further enhancements for patients and health systems.
